# Thermo-Plasmonics for Localized Graphitization and Welding of Polymeric Nanofibers

**DOI:** 10.3390/ma7010323

**Published:** 2014-01-13

**Authors:** Ahnaf Usman Zillohu, Nisreen Alissawi, Ramzy Abdelaziz, Mady Elbahri

**Affiliations:** 1 Institute of Polymer Research, Nanochemistry and Nanoengineering, Helmholtz-Zentrum Geesthacht, Max-Planck-Strasse 1, 21502 Geesthacht, Germany; E-Mails: usman.zillohu@hzg.de; 2 Nanochemistry and Nanoengineering, Faculty of Engineering, Institute for Materials Science, University of Kiel, Kaiserstrasse 2, 24143 Kiel, Germany; E-Mails: nal@tf.uni-kiel.de (N.A.); rba@tf.uni-kiel.de (R.A.)

**Keywords:** electrospinning, plasmonic heat, graphitization, welding

## Abstract

There is a growing interest in modulating the temperature under the illumination of light. As a heat source, metal nanoparticles (NPs) have played an important role to pave the way for a new branch of plasmonics, *i.e.*, thermo-plasmonics. While thermo-plasmonics have been well established in photo-thermal therapy, it has received comparatively less attention in materials science and chemistry. Here, we demonstrate the first proof of concept experiment of local chemistry and graphitization of metalized polymeric nanofibers through thermo-plasmonic effect. In particular, by tuning the plasmonic absorption of the nanohybrid through a change in the thickness of the deposited silver film on the fibers, the thermo-plasmonic effect can be adjusted in such a way that high enough temperature is generated enabling local welding and graphitization of the polymeric nanofibers.

## 1. Introduction

The use of plasmonic heat in medical therapy is well established and a variety of applications of this phenomenon have already been studied and reviewed [1], including targeted hyperthermia, destruction of tumor cells and drug release. In materials science, although its effectiveness has been shown by various applications ranging from catalysis [2], to preparation of transparent conductors for solar cells [3], or fabrication of sensors based on surface enhanced Raman [4], but its full potential still remains to be explored.

The properties of nanohybrids can be tailored by controlling the chemical composition, size, shape as well as the volume fraction and the distribution of the nanoscale particles [5,6]. Interaction of light with NPs results in its absorption and scattering, where absorption dominates scattering for a particles size smaller than 100 nm [7]. The light absorption increases with increasing particle size by a power of three and is given by [7]:

$$\sigma_{abs}=\frac{\pi^{2}}{\lambda }R^{3}\text{Im}\{(\varepsilon_{p}-\varepsilon_{s})/\left(\varepsilon_{p}+2\varepsilon_{s}\right)\}$$ (1)

where σ*_abs_* is the absorption cross-section and *R* is radius of the NPs; ε*_p_* and ε*_s_* are the wavelength dependent complex dielectric function of metal NPs and the surrounding matrix respectively. The light is absorbed by NPs in the form of coherent oscillations of their electrons. These oscillations, called plasmons, can be of propagating nature as in the case of thin films, or confined to individual NPs or clusters. The dissipation of these oscillations results in heating of NPs and their surrounding environment.

Plasmonic heat generation by pulsed laser (usually nano to femtosecond pulse widths) irradiation has been well studied [8–10]. The physical picture that arises from these studies showed that, because of low optical quantum yield of metallic NPs, the total heat generated can be roughly equated to the optical energy absorbed. The energy absorbed by the electrons puts the electron gas into non-Fermi electron distribution which thermalizes to equilibrium by electron-electron scattering. At this stage the electron gas may have a temperature of up to several thousand kelvin. The electron gas then cools by electron-phonon coupling with the NP lattice. The NP then cools by heat dissipation to the surrounding matrix through phonon-phonon interaction.

Heat transfer equations that govern the temperature *T* of a sphere and its surroundings are [11,12]:

$$\frac{\partial T_{p}}{\partial t}=\frac{\alpha_{p}}{r}\frac{\partial^{2}}{\partial r^{2}}[rT_{p}(r,t)]$$ (2)

$$\frac{\partial T_{s}}{\partial t}=\frac{\alpha_{s}}{r}\frac{\partial^{2}}{\partial r^{2}}[rT_{s}(r,t)]$$ (3)

where the subscript *p* refers to the particles, *s* refers to their surrounding, α is the thermal diffusivity of the particle or the surroundings and *r* is the distance away from the center of the nanoparticle. In fact α is related to thermal conductivity *k*, by the relation, α = *k*/ρ*C_p_* where ρ and *C_p_* are density and specific heat capacity, respectively. The Equations (2) and (3) were coupled with Maxwell’s equations and numerically solved [13] to show that in the steady state regime, the local temperature rise, *∆T*(*r*), around a single NP is described by the following Equation:

$$\Delta T(r)=\frac{V_{p}Q}{4\pi k_{s}r}$$ (4)

where *k_s_* is the thermal conductivity of the surrounding medium, *V_p_* is the volume of the NP and *Q* is the heat generated in the nanoparticles. Note that Equation (4) is applicable only in case where the distance *r* is greater than radius of the NP.

If the incident light wavelength is much larger than the radius of the NP then the heat generation would be [14]:

$$Q=\frac{\omega }{8\pi }E_{0}^{2}{\left|\frac{3\varepsilon_{s}}{2\varepsilon_{s}+\varepsilon_{p}}\right|}^{2}\text{Im}\varepsilon_{p}$$ (5)

where *E*_0_ is the amplitude of incident radiation and ω is its angular frequency, ε*_s_* is the dielectric constant of the surrounding environment of NP and ε*_p_* and Im ε*_p_* are the real and the imaginary parts of the dielectric constant of the NP, respectively.

From Equations (1) and (5) it is evident that by tuning the particle size and absorption coefficient (which depends on the type of metal), the total light absorption and its conversion to heat can be controlled. The temperature rise of the surrounding not only depends upon *Q* but also on *V_p_*, as shown in Equation (4), and thus we expect from a bigger particle to raise the temperature of its surrounding to a higher level than a smaller particle would do. The generated heat can be high enough to cause melting or even decomposition of the surrounding. Since the energy from the incident light is confined to individual NPs therefore the heating effect is also localized to their near vicinity. This not only eliminates the danger of collateral damage by undesired heating but in fact is useful in physical and chemical re-structuring and patterning of matter at submicron scale [15–17].

Recently we introduced the possibility of NPs incorporation into a polymer film upon low energy laser irradiation to fabricate a metal-polymer nanocomposite [17]. We showed that selective and precise embedding of Ag NPs in Polyvinylidene fluoride (PVDF) polymeric matrix can be carried out for *in-situ* fabrication of nanocomposites in different forms. That included controlled patterning, writing, defect healing and welding in a controlled manner along with crystallinity control through light irradiation. Indeed, the silver NPs were activated using laser irradiation of only 2–10 mW, causing absorption of photons and the transfer of resulting heat from the NPs to the surrounding polymer matrix. The local heating disrupted the polymer matrix and allowed softening and pre-melting of the subsurface layer of the polymer foil with resulting embedment of Ag NPs in it. Here we introduce our recent progress with the thermo-plasmonics, demonstrating the first experimental proof of light induced local heating and graphitization of polymer-fibers decorated with Ag NPs.

PVDF is known for its piezoelectric properties besides high chemical stability and good mechanical properties. Graphitization of PVDF electrospun mats have earlier been demonstrated by conventional heating (800 °C to 1800 °C) in the presence of iron(III) acetylacetonate catalyst [18]. Graphitized nanofibers are a potential candidate for hydrogen storage [18] or as electrodes for supercapacitors [19], to name a few applications. In this work PVDF electropsun nanofibers were sputter coated with silver NPs and subsequently exposed to laser in a Raman microscope. Graphitization of polymer nanofibers using thermo-plasmonic concept requires significant increase in the temperature of NPs sputtered over them. By tuning the size, shape and interparticle distance, we expect a huge quantity of heat to be generated by the NPs. We demonstrate that the temperature rise is actually high enough to weld the fibers together and even to graphitize the polymer. The novelty of the work presented here is that individual fiber can be graphitized “locally” by plasmonically generated heat.

## 2. Results and Discussion

**Figure 1.**
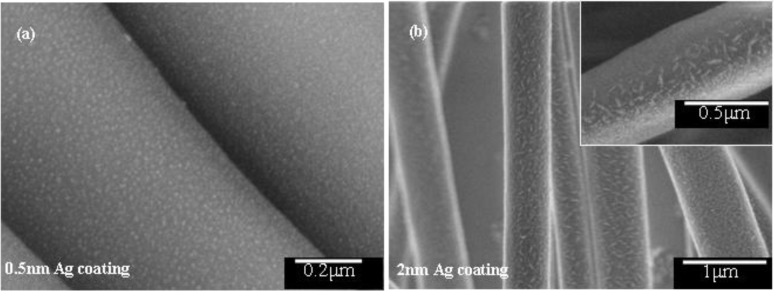
Variation of silver nanoparticles’ shape and size with increasing sputtering thickness (white particles on darker fibers). (**a**) 0.5 nm thick silver coating resulted in isolated round particles; (**b**) 2 nm thick coating resulted in elongated agglomerates (inset is a closer view). The micrographs were made by mixing the signal from backscattered electron and secondary electron detectors.

Electrospinning is a fast and easy method of producing fibers from a variety of polymer solutions. For beadless, uniform fibers it is important to have a solution of sufficiently high viscosity. The PVDF grade used for electrospinning in the present study gives a high viscosity solution of up to 8000 cps [20]. Moreover, N,N-Dimethylformamide (DMF) was used as the solvent, which has a high dipole moment [21] of 3.8 Debye and high conductivity [21] of around 10.9 μS/cm which improves charge carrying capacity of the jet. As a result a thin mat of uniform, beadless fibers in submicron size was collected. The fibers were then sputter coated with silver to various layer thickness. Regarding the distribution of silver nanoparticles on the fibers, with the help of scanning electron microscope (SEM), it was found that the NPs almost completely filled the surface and generated a hierarchical structure on top of the fibers (Figure 1). Obviously, the smallest particles of silver were observed for 0.5 nm thick coating and were about 12 nm in size and spherical in shape (Figure 1). With increasing coating thickness, not only that the particles’ size increased but they also got elongated because of joining of incoming neighboring particles. This resulted in a vast size distribution for 2 nm thick coating and up; ranging from small spherical particles to elongated particles with longer axis as big as 150 nm (Figure 1).

**Figure 2.**
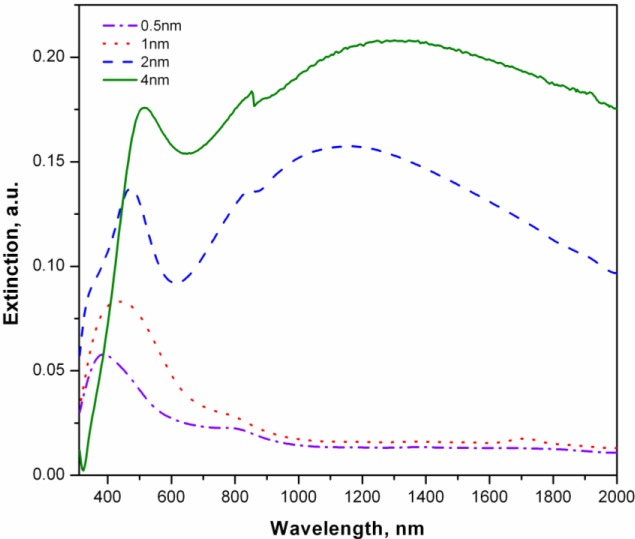
UV-Vis spectrum of “polyvinylidene fluoride (PVDF) fiber-Ag composite” with increasing thickness of silver coat (0.5 to 4 nm).

The change in silver coating thickness and a corresponding change in particle size, shape and the inter-particle distance, effects the plasmon resonance wavelength of the system. The plasmon resonance wavelength associated with NPs increases with their size due to retardation effect [22,23]. This is evident in the UV-Vis results (Figure 2), where the increase in layer thickness from 0.5 to 1 nm not only increased the extinction maxima but the maxima also shifted to the longer wavelength.

This red shift stems not only from the size increase but also from near field coupling [24,25] of growing NPs, as they approach each other. By the SEM observation (Figure 1), it was found that for 2 nm coating thickness (and beyond) the NPs start to touch each other with consequent elongation in overall shape. The elongation of the NPs also results in further red shift [26] but more interestingly we observe multimode plasmon (plasmon splitting) and extinction in the near IR range. By adding more Ag up to 4 nm, a red shift for both peaks (visible and NIR) along with broadening was observed. The appearance of multimode plasmon can be associated to the hybridization of individual plasmon mode as the approaching nanoparticles enter from dielectric proximity to conductively coupled regime [27]. Presence of a super strong concentration of electric field at the point joining two NPs has been already shown by Atay *et al.* [27]. Indeed, the dissipation of this field into lattice phonons is expected to result in a huge local heat, as mentioned previously. To examine this hypothesis we exposed the fibers to green laser (532 nm wavelength) of 20 mW power, in the Raman microscope.

**Figure 3.**
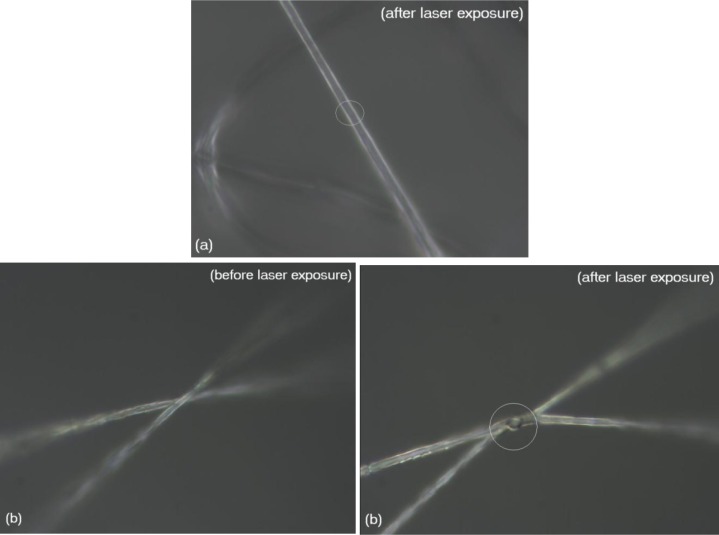
Effect of 532 nm, 20 mW laser on fibers. (**a**) Uncoated fibers showed no melting on exposure to laser (encircled area); (**b**) Fibers coated with 2 nm Ag showed welding/graphitization on exposure to laser.

**Figure 4.**
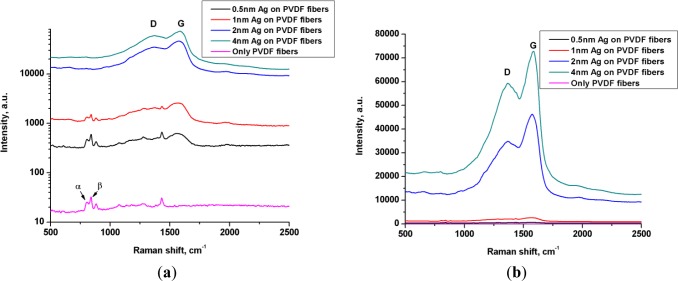
(**a**) Raman spectrum of “PVDF fiber-silver NPs” composite with different silver layer thickness (0.5 to 4 nm); (**b**) Same data as in (**a**), but plotted on “linear” scale to highlight the presence of D and G bands.

In the case of uncoated fibers, no melting or decomposition was observed, as shown by the optical micrograph in Figure 3a. Here, it is clear that the portion of the fiber that was exposed to the laser showed no signs of melting/graphitization. However, in the case of fibers with 2 nm thick silver coating, the junction point melted and fused the fibers together, on exposure to the laser (Figure 3b). This indicates that the plasmonic heat generated by silver nanoparticles was much higher than the melting point of the polymer, which is around 172 °C [20]. Interestingly, the “joint” also showed blackening which turned out to be local graphitization as seen by the Raman analysis (Figure 4).

The Raman signal scattered by uncoated fibers clearly showed the presence of PVDF as depicted by its finger print peaks at 795 cm^−1^ for alpha conformation and 839 cm^−1^ corresponding to beta conformation [28] (Figure 4a).

It is worth noting that there was no evidence of graphitic structure in the Raman spectrum of uncoated fibers. This was supported by the visual observation mentioned above, that the uncoated fibers could sustain laser without melting or graphitization.

With increasing thickness of silver coating, an enhancement in the Raman signal was observed meaning stronger interaction of light with the system (Figure 4a). This was in accordance with that observed in the UV-Vis analysis (Figure 2), where an overall increase in absorption of light, including that in the wavelength region of the green laser, was observed with increasing thickness of the silver coating. More importantly, the Raman signal scattered by the fibers coated with 0.5 and 1 nm silver, still confirmed the integrity of the chemical structure of polymer as seen by the presence of peaks corresponding to α and β phase of PVDF. On the other hand, two additional peaks also appeared, belonging to D and G bands of graphite [20,29,30] at 1368 cm^−1^ and 1580 cm^−1^ respectively (Figure 4b). The intensity of these bands got stronger in the case of 2 nm and 4 nm silver coating and indeed, the PVDF peaks were diminished and were replaced by strong graphite peaks indicating a complete graphitization of the polymer. Raman analysis during graphitization with 20 mW laser and its reanalysis with a lower power laser (0.2 mW) shows persistent presence of graphite and absence of PVDF, indicating a permanent conversion (data not shown).

While this finding is the main scientific message in this communication, we believe that work is still needed to explore the chemical transformation as well as the actual temperature rise, which is not a simple task. Nevertheless, we believe that our finding of “thermo-plasmonic based local graphitization” serves only as an example and would open a new avenue in the possibilities of local chemistry and structural control. The applications of such a polymer-graphite hybrid would be diverse, for example, it can be conductive for electrical contacts over a part of its length while piezoelectric over the rest of the length.

## 3. Experimental Section

PVDF (Kynar HSV 900, Arkema Inc., Pennsylvania, PA, USA) solution for electrospinning was prepared by dissolving 11 wt% PVDF in N,N-Dimethylformamide (DMF) (Sigma-Aldrich, Steinheim, Germany). The solution was then pushed through a needle that had 0.8 mm orifice diameter. A target plate, that was covered with glass slides was placed at a distance of 15 cm from the needle and served as collector. The needle was attached to a high voltage (15 kV) supply and target plate was attached to ground potential. As a result of electrostatic force, the polymer coming out of the needle was accelerated and stretched in the form of nanofibers which were collected on the target plate. These fibers were then coated with silver to a thickness of 0.5 nm, 1 nm, 2 nm and 4 nm by physical vapor deposition process, the thickness being controlled with the help of a quartz microbalance. The UV-Vis characterization of the coated fibers was done with “Lambda 900” UV-Vis/IR spectroscope (PerkinElmer, Rodgau, Germany) by normalizing the background to uncoated PVDF fibers. Raman analysis and welding/graphitization was performed on Bruker confocal Raman microscope (Bruker Optik GmbH, Ettlingen, Germany) equipped with 532 nm continuous wave laser. SEM imaging of the silver coated fibers was performed on LEO Gemini 1550 VP from Zeiss with field emission cathode (Carl Zeiss Microscopy GmbH, Oberkochen, Germany).

## 4. Conclusions

Electrospun PVDF fibers were sputter coated with silver to different film thicknesses ranging from 0.5 to 4 nm. SEM and UV-Vis analysis showed that with increasing coating thickness there was an increase in plasmonic absorption and a red shift in extinction maxima. The heating effect of thermo-plasmonic increased with increasing coating thickness and was ultimately sufficient for a complete local conversion of the polymeric fiber to graphitic fiber.
